# Pelvic floor and sexual health outcomes after cesarean hysterectomy for placenta accreta spectrum

**DOI:** 10.1002/pmf2.70134

**Published:** 2025-10-18

**Authors:** Austin Oberlin, Meghan Angley, Helai Hesham, Fady Khoury Collado, Eve Overton, John G. Ilagan, Laurence E. Ring, Alexandre Buckley De Meritens, Mirella Mourad

**Affiliations:** ^1^ Department of Obstetrics and Gynecology Columbia University Irving Medical Center New York New York USA; ^2^ Division of Gynecologic Specialty Surgery Department of Obstetrics and Gynecology Columbia University Irving Medical Center New York New York USA; ^3^ Division of Gynecologic Oncology Department of Obstetrics and Gynecology Columbia University Irving Medical Center New York New York USA; ^4^ Division of Maternal‐Fetal Medicine Department of Obstetrics and Gynecology Columbia University Irving Medical Center New York New York USA; ^5^ Department of Anesthesiology Columbia University Irving Medical Center New York New York USA

**Keywords:** cesarean hysterectomy, dyspareunia, pelvic organ prolapse, placenta accreta spectrum, urinary incontinence

## Abstract

**Objective(s):**

To estimate the incidence of urinary incontinence, pelvic organ prolapse, and sexual dysfunction in a cohort of patients who had undergone hysterectomy for placenta accreta spectrum (PAS). Additionally, to understand whether certain surgical factors contributed to these outcomes.

**Methods:**

This was a retrospective cohort study of patients who underwent a cesarean hysterectomy for PAS between January 2018 and August 2023 at a single urban academic institution. Patients were recruited 6 months or more following surgery performed by the dedicated accreta team. The survey consisted of validated questionnaires (Pelvic Floor Distress Inventory and Pelvic Organ Prolapse/Urinary Incontinence Sexual Questionnaire), and clinical data were abstracted from the medical record. Patients who reported pre‐existing incontinence or who were not sexually active were excluded from these respective outcomes. Two‐sided tests of significance were used with an alpha level of 0.05. There was no adjustment for multiple comparisons.

**Results:**

During the study period, 100 patients underwent a hysterectomy for PAS of which 64 completed the survey. Participants had a median age of 36 at the time of surgery and a median of 2 prior cesarean deliveries. The majority of cases were scheduled (*n* = 44, 68.8%) and underwent a supracervical hysterectomy (*n* = 40, 62.5%). Cystotomy occurred in 12.5% (*n* = 8/64) of cases. Following hysterectomy, approximately 31% (*n* = 20/64) of participants had at least one symptom of prolapse, 39.3% (*n* = 24/61) had symptoms of stress urinary incontinence, and 23.0% (*n* = 14/61) had symptoms of urge incontinence. Twenty percent of patients (*n* = 13/64) reported chronic pelvic pain, while 48.0% (*n* = 25/52) reported at least occasional dyspareunia. Rates of stress incontinence (*p* = 0.048) and pelvic pain (*p* = 0.047) were reported more frequently in patients who had experienced cystotomy. There was no difference in outcomes when comparing the type of hysterectomy (i.e., total vs. supracervical) or the urgency of the surgical case.

**Conclusion:**

In this cohort of patients who underwent hysterectomy for placenta accreta, there was a high rate of symptoms of prolapse, urinary incontinence, pelvic pain, and dyspareunia, especially in those with intraoperative bladder injury. These results should be used to counsel patients prior to surgery. Placenta accreta centers of excellence should consider offering referral to urogynecology postoperatively for all patients, and especially for those with intraoperative bladder injury.

**Abbreviations**
PAS: placenta accreta spectrum
POP: pelvic organ prolapse
SUI: stress urinary incontinence
UUI: urge urinary incontinence

## 1 INTRODUCTION

Placenta accreta spectrum (PAS), defined as the abnormal adherence of the placenta to the uterine wall during pregnancy, remains a relatively understudied entity. Recent estimates suggest that PAS occurs in approximately 1 in every 313 patients undergoing cesarean delivery [1]. Much research in the past two decades has focused on identifying risk factors, comparing treatment techniques, and estimating short‐term outcomes for PAS; however, less is known about the longer‐term impact [1, 2, 3, 4]. Grover et al. [5] reported on long‐term health outcomes and quality of life up to 3 years after pregnancy complicated by PAS. They found that pregnant people with PAS were more likely to report decreased quality of life and worse health outcomes years after treatment. Another study, Bartels et al. [6], examined quality of life scores in patients with a history of PAS and found over 50% met criteria for sexual dysfunction. Finally, Crocetto et al. [7] found that 21% of patients reported urinary incontinence (UI) after surgery for PAS, a significant increase over controls. However, this study did not report on other pelvic floor or sexual health outcomes.

Both pregnancy and hysterectomy for other indications increase a person's risk of UI, pelvic organ prolapse (POP), and other adverse urogynecologic outcomes [8, 9]. Cesarean delivery may not be as protective against these outcomes as has previously been thought [10]. The pathophysiology of PAS (i.e., thinning of the myometrium, compression of local structures, neovascularization) as well as changes in the pelvic anatomy related to surgery (i.e., removal of the uterus, alteration of vascular perfusion, or denervation of structures) could all theoretically impact a person's risk for these outcomes [11, 12].

The primary aim of this study was to explore the incidence of UI, POP, and sexual dysfunction in a cohort of pregnant people who have undergone hysterectomy for PAS. Our secondary aim was to understand whether specific surgical factors contributed to these outcomes.

## 2 METHODS

We performed a retrospective cohort study of patients who underwent a cesarean hysterectomy for pathologically confirmed PAS at a single urban academic institution. All patients who underwent surgery from January 2018 to August 2023 were recruited by their primary obstetrician to complete a survey about their pelvic and sexual health after surgery. Patients with surgery prior to 2018 were not included, as there was a higher degree of heterogeneity in the surgical approach (e.g., the use of IR‐guided balloon catheterization) prior to this time. During the study period, no patient underwent pre‐ or intraoperative vascular catheterization, all patients had indwelling ureteral catheters in addition to the Foley catheter and all were in dorsal lithotomy position. In addition to cross‐matched blood products, a cell‐saver device was used during all cases. The standard of our placenta accreta team was to perform a supracervical hysterectomy unless the surgeon deemed removal of the cervix necessary to reduce blood loss or complete the procedure. This approach has been validated by other practitioners [13]. Intentional cystotomy, as has been described by other authors, was not performed [14].

Patients were not recruited until at least 6 months after surgery, so as to identify symptoms that persisted beyond the acute recovery period. Surveys were initially sent in April 2023, with follow‐up on those who had not completed the survey through January 2024. Thus, patients completed the survey anywhere from 6 months to 4 years after surgery. Patients provided informed consent including access to protected health information, after which they completed the electronic survey through the REDCap survey database at their own convenience [15]. After completion of the survey, key medical and surgical history were extracted from the patient's medical record. The study was reviewed and approved by the local Institutional Review Board.

We chose self‐reported, validated measures of pelvic floor and sexual health. The Pelvic Floor Distress Inventory (PFDI) is a patient questionnaire that has been validated to measure pelvic floor dysfunction, including in the postpartum period [16, 17]. It consists of 20 questions with a Likert‐scale format. For example, “Do you usually experience urine leakage related to laughing, coughing, or sneezing? If Yes, how much does it bother you? Not at all, somewhat, moderately, or quite a bit.” The PFDI consists of six questions related to POP, eight related to colorectal or anal distress, and six related to urinary distress or incontinence. The Pelvic Organ Prolapse/Urinary Incontinence Sexual Questionnaire (PISQ‐IR) is a validated questionnaire developed by the International Urogynecological Association to measure sexual function in people with POP [18]. This questionnaire consists of 20 questions in a Likert scale format. Patients were additionally asked to self‐report a history of POP, UI, diabetes, asthma, or smoking. A complete copy of the survey can be found in Supporting Information 1.

Raw data files were extracted from the REDCap database and de‐identified. They were then imported into SAS 9.4 (Cary, NC) for analysis. Patients who reported pre‐existing stress urinary incontinence (SUI) or urge urinary incontinence (UUI) prior to pregnancy or who were not sexually active were excluded from these respective outcomes. There were no missing data, as patients were required to complete each question on the survey. Two‐sided tests of significance (chi‐square or Fisher's exact test, where appropriate) were used with an alpha level of 0.05 used to determine significance. There was no adjustment for multiple comparisons. A multivariate analysis was planned a priori comparing risk of POP, SUI, UUI, pelvic pain or dyspareunia between those with cystotomy versus without a cystotomy and covariates including age, parity, and body mass index (BMI).

## 3 RESULTS

**FIGURE 1:**
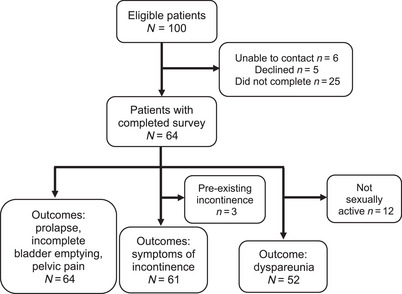
Flow diagram of survey participants.

From January 2018 to August 2023, 100 patients underwent surgery for PAS at our institution. Of these 100 patients, 64 completed the survey and were included in the analysis. Three patients reported pre‐existing UI, while twelve patients were not sexually active and were excluded from these respective outcomes (Figure 1). Characteristics of the participants who completed the survey are summarized in Table 1. Patients had a median of 2 prior cesarean deliveries prior to the index pregnancy, and maternal pre‐pregnancy BMI of 30.8 (inter‐quartile range [IQR]: 27–36). The standard practice at our institution during the study period was to perform a supracervical hysterectomy unless removal of the cervix was deemed necessary to control surgical bleeding. Approximately two‐thirds of patients underwent a supracervical hysterectomy. The majority of procedures were performed as scheduled, though 31.3% occurred emergently. The only surgical complication noted at the time of delivery was unintentional cystotomy, which occurred in 12.5% (*n* = 8/64) of patients. No intentional cystotomies were performed. All injuries were noted at the time of surgery, repaired with the help of a urologist, and treated postoperatively per usual standard of care (i.e., Foley catheter for 7–14 days, CT urogram before catheter removal).

**TABLE 1:** Patient and surgical characteristics (*N* = 64).

	Median (IQR)
Maternal age	36 (33–39)
Parity	2 (1–4)
Prior cesarean deliveries	2 (1–2)
Gestational age (at delivery)	34.4 (33.2–35.4)
Maternal BMI	30.8 (27.0–36.0)

	*N* (%)
Hysterectomy type	
Total	24 (37.5)
Supracervical	40 (62.5)
Surgery setting	
Scheduled	44 (68.8)
Emergent	20 (31.3)
Blood transfusion	
Yes	38 (59.4)
No	26 (40.6)
ICU admission	
Yes	3 (4.7)
No	61 (95.3)
Cystotomy/bladder injury	
Yes	8 (12.5)
No	56 (87.5)

Abbreviations: BMI, body mass index; ICU, intensive care unit; IQR, inter‐quartile range.

The most commonly reported symptom more than 6 months after surgery was dyspareunia (*n* = 25/52, 48.1%; Table 2). Next most common was UI, with 39.3% reporting symptoms of SUI (*n* = 24/61) and 23.0% reporting symptoms of UUI (*n* = 14/61). Approximately 30% of patients reported at least one symptom of POP, most commonly pressure in the lower abdomen or heaviness/dullness in the pelvic area. Only 3% reported feeling a bulge or something falling out of the vagina. Chronic pelvic pain was reported in 20.3% (*n* = 13/64) of patients. Rectal/Anal symptoms were rare and are not reported here.

**TABLE 2:** Self‐reported symptoms of pelvic floor and sexual dysfunction after cesarean hysterectomy, with subgroup analysis for bladder injury, type of hysterectomy, and urgency of the surgical case.

		Bladder injury			Type of hysterectomy			Urgency of surgical case		
	Total *n*/*N* (%)	Yes *n*/*N* (%)	No *n*/*N* (%)	*p* value^a^	SCH *n*/*N* (%)	Total *n*/*N* (%)	*p* value^a^	Scheduled *n*/*N* (%)	Emergent *n*/*N* (%)	*p* value^a^
Symptoms of POP	20/64 (31.8)	5/8 (62.5)	15/56 (26.8)	0.10	12/40 (30.0)	8/24 (33.3)	0.78	12/44 (27.3)	8/20 (40.0)	0.31
Self‐reported SUI^b^	24/61 (39.3)	6/8 (75)	18/53 (34)	<0.05	16/37 (43.2)	8/24 (33.3)	0.44	14/41 (34.2)	10/20 (50)	0.23
Self‐reported UUI^b^	14/61 (23.0)	3/8 (37.5)	11/53 (20.8)	0.37	6/37 (16.2)	8/24 (33.3)	0.12	9/41 (22)	5/20 (25)	0.99
Incomplete bladder emptying	8/64 (12.5)	2/8 (25)	6/56 (10.7)	0.26	4/40 (10)	4/24 (16.7)	0.46	4/44 (9.1)	4/20 (20)	0.24
Pelvic pain	13/64 (20.3)	4/8 (50)	9/56 (16.1)	<0.05	8/40 (20)	5/24 (20.8)	0.99	6/44 (13.6)	7/20 (35)	0.09
Dyspareunia^c^	25/52 (48.1)	5/6 (83.3)	20/46 (43.5)	0.09	16/33 (48.5)	9/19 (47.4)	0.94	16/37 (43.2)	9/15 (60)	0.27

Abbreviations: POP, pelvic organ prolapse; SCH, supracervical hysterectomy; SUI, stress urinary incontinence; UUI, urge urinary incontinence.

^a^ *p* value calculated using the chi‐square test or Fisher's exact test when appropriate.

^b^ Excluding those with a history of incontinence (*n* = 3).

^c^ Excluding those who are not sexually active (*n* = 12).

Patients reported that these symptoms were highly bothersome and had an impact on their lives. For those with symptoms of SUI, 45.8% (*n* = 11/24) said the symptoms were moderately or quite bothersome. For those with symptoms of pelvic pain, 69.2% (*n* = 9/13) found it bothersome. Finally, of the 14.1% (*n* = 9/64) who reported not being sexually active, four out of nine reported avoiding sex due to symptoms of prolapse, incontinence, or pelvic pain.

**FIGURE 2:**
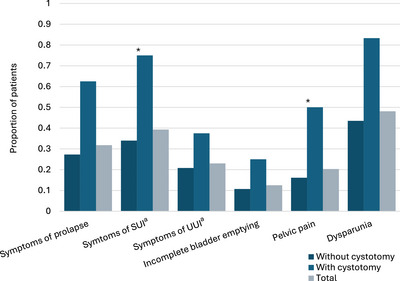
Self‐reported symptoms of pelvic floor and sexual dysfunction, stratified by intraoperative cystotomy. SUI, stress urinary incontinence; UUI, urge urinary incontinence. ^a^Those with self‐reported history of urinary incontinence were excluded (*n* = 3). ^*^Difference was statistically significant (*p* < 0.05) using the chi‐square test or Fisher's exact test when appropriate.

To determine whether surgical factors influenced patient outcomes, a univariate analysis was performed. Symptoms of prolapse, SUI, UUI, incomplete bladder emptying, pelvic pain, and dyspareunia were all higher among patients who had an intraoperative cystotomy. This association was statistically significant for symptoms of SUI and pelvic pain (Figure 2). Due to the low number of observations in each group, a multivariate analysis was not performed. There was no association between any of these symptoms and the type of hysterectomy performed (i.e., supracervical or total) nor in the urgency of the surgical case (i.e., scheduled vs. emergent; Table 2).

Finally, though we did not follow patients longitudinally, we compared rates of reported symptoms between those less than 1 year, between 1 and 2 years, and more than 2 years after hysterectomy at the time of survey completion. Symptoms of prolapse, SUI, UUI, incomplete bladder emptying, and dyspareunia were not significantly different depending on the time since their surgery. None of the reported symptoms showed a trend in the rate, either higher or lower, over time (Supporting Information 2).

## 4 DISCUSSION

Limited research exists on patient outcomes treated for PAS beyond the immediate postoperative period [5, 6, 7]. For patients who undergo surgery for PAS, both pregnancy and hysterectomy may put them at risk for long‐term pelvic floor and sexual dysfunction. The pathophysiology of PAS (i.e., thinning of the myometrium, compression of local structures, neovascularization) may increase this risk. Moreover, disruption of pelvic innervation, vasculature, and supporting ligaments during a complex surgery could further contribute to dysfunction. In this study, of patients who have undergone hysterectomy for pathologically confirmed PAS, we found a high prevalence of symptoms of POP, UI, chronic pelvic pain, and dyspareunia.

Although we did not include a control group, comparing our findings to related groups may help provide context. In one prior study, the risk of UI after hysterectomy for PAS was 21%, which was significantly higher than in a control group who underwent planned cesarean delivery [7]. Notably, rates of SUI were higher than rates of UUI, which is consistent with our findings. However, here we find that cystotomy was associated with a higher rate of SUI. In concordance with these findings, a systematic review of cystotomy during obstetric or gynecologic surgery found that it may be associated with postoperative UI [19]. The prevalence of incontinence in postpartum women in general is around 30%, notably lower than in our current study [20].

In our current study, dyspareunia was reported in nearly half of the patients surveyed who reported being sexually active. This is higher than rates reported in the postpartum period of otherwise uncomplicated pregnancies; one meta‐analysis estimated a rate of dyspareunia of 22% at 6–12 months postpartum [21]. Similarly, our study found higher rates of pelvic pain than have been reported in the literature for patients after cesarean delivery. A meta‐analysis found that only 1.3% of patients still report pelvic pain at least 12 months after cesarean delivery [22]. Importantly, in our study, a large proportion of patients who reported these symptoms found them bothersome or led them to avoidance of sexual activity. Finally, the rates of symptoms of SUI, UUI, incomplete bladder emptying, and dyspareunia remain high even in those who completed the survey more than 2 years after surgery (Supporting Information 2). Although our study was not longitudinal, these symptoms remain prevalent regardless of the time since surgery.

Our findings also identify intraoperative cystotomy as an important risk factor for pelvic floor and sexual dysfunction. Those with an intraoperative bladder injury had a trend toward higher rates of symptoms of POP, UI, incomplete bladder emptying, pelvic pain, and dyspareunia. Although this association only reached statistical significance for symptoms of SUI and pelvic pain, clinicians should be vigilant for any symptom of pelvic floor dysfunction in patients with a cystotomy during surgery for PAS. Other surgical factors were not associated with these symptoms in this cohort. Supracervical hysterectomy, which, for abdominal hysterectomy, has been associated with higher rates of UI but similar rates of prolapse to total hysterectomy, was not associated with any difference in outcomes in this study [23].

Our study has limitations: outcomes are based on patient self‐reported symptoms rather than objective clinical observation, and survey responses may be subject to sampling or response bias. We chose not to include a control group, as the primary aim was to define rates of patient outcomes among those undergoing hysterectomy for PAS rather than to measure relative risk compared with other obstetric populations. This limits our ability to make conclusions about relative risks, though it does not prevent clinicians from using these absolute risk estimates to counsel patients. Since hysterectomy was the standard of care at our institution during this time period, no alternative treatment group was available as a control. Time from surgery to survey follow‐up ranged widely from 6 months to 4 years, which introduces a degree of heterogeneity into the cohort, though we did not find differences in symptom prevalence over time (Supporting Information 2). Our findings are strengthened by a large number of cases from a single institution during a time period when the surgical approach was standardized, which reduces the impact of confounding variables. Our survey response rate of >60% may reduce the incidence of sampling bias, though we cannot eliminate the possibility that those with symptoms may be more likely to complete the survey than those without.

In conclusion, patients who undergo cesarean hysterectomy for PAS frequently experience pelvic floor and sexual dysfunction, especially in those with intraoperative bladder injury. A larger multicenter study, with higher power to detect differences in those with cystotomy, may help inform whether the practice of intentional cystotomy carries a higher risk of these long‐term outcomes. Practitioners should consider incorporating these findings into preoperative counseling for patients with PAS, both to adequately inform them and to help set expectations for recovery. PAS centers of excellence should consider incorporating routine referral to urogynecology and pelvic floor physical therapy for patients after surgery.

## AUTHOR CONTRIBUTIONS

Austin Oberlin, Helai Hesham, Fady Khoury Collado, Eve Overton, John G. Ilagan, Laurence E. Ring, Alexandre Buckley De Meritens, Mirella Mourad were involved in the conceptualization and design of the study. Austin Oberlin, Mirella Mourad, and Fady Khoury Collado were involved in participant recruitment. Meghan Angley and Austin Oberlin performed the statistical plan and analysis. Austin Oberlin drafted the manuscript, and all authors were involved in writing and approved the final manuscript.

## CONFLICT OF INTEREST STATEMENT

The authors declare no conflicts of interest.

## FUNDING INFORMATION

Funds for the study were provided from an unaffiliated fund managed by Dr. Mirella Mourad. No external funders were involved in the design, analysis or publication of this manuscript.

## Supporting information



Supporting Information

Supporting Information

## Data Availability

Original data are available upon request to the corresponding author.
