# Supplementary material for: Pelvic floor and sexual health outcomes after cesarean hysterectomy for placenta accreta spectrum
Source: Pregnancy (Hoboken). 2025 Oct 18;1(6):e70134. doi: 10.1002/pmf2.70134 (PMC13344666; doi:10.1002/pmf2.70134)
Supplement: Supplementary file 1 [file PMF2-1-e70134-s001.pdf]

PFDI-20

Have you ever been diagnosed with any of these conditions BEFORE your pregnancy:	☐ Pelvic organ prolapse<br>☐ Stress urinary incontinence (leakage of urine when you cough, laugh or sneeze)<br>☐ Urge urinary incontinence (leakage of urine due to a sudden urge to void)<br>☐ Mixed urinary incontinence (A combination of stress and urge urinary incontinence)<br>☐ None of the above
Have you ever been diagnosed with diabetes?	◯ Type 1 Diabetes<br>◯ Type 2 Diabetes<br>◯ Gestational Diabetes<br>◯ None of the above
Have you ever been diagnosed with asthma?	◯ No<br>◯ Yes
Do you smoke cigarettes or tobacco?	◯ No<br>◯ Yes

Pelvic Floor Distress Inventory Questionnaire - Short Form 20

Please answer all of the questions in the following survey. These questions will ask you if you have certain bowel, bladder or pelvic symptoms and, if you do, how much they bother you. Answer each question by making the appropriate selection. If you are unsure about how to answer, please give the best answer you can. While answering these questions, please consider your symptoms over the last 3 months.

1. Do you usually experience pressure in the lower abdomen?	◯ Yes<br>◯ No
If Yes, how much does it bother you?	◯ Not at all<br>◯ Somewhat<br>◯ Moderately<br>◯ Quite a bit
2. Do you usually experience heaviness or dullness in the pelvic area?	◯ Yes<br>◯ No
If Yes, how much does it bother you?	◯ Not at all<br>◯ Somewhat<br>◯ Moderately<br>◯ Quite a bit
3. Do you usually have a bulge or something falling out that you can see or feel in your vaginal area?	◯ Yes<br>◯ No
If Yes, how much does it bother you?	◯ Not at all<br>◯ Somewhat<br>◯ Moderately<br>◯ Quite a bit

4. Do you ever have to push on the vagina or around the rectum to have or complete a bowel movement?	◯ Yes<br>◯ No
If Yes, how much does it bother you?	◯ Not at all<br>◯ Somewhat<br>◯ Moderately<br>◯ Quite a bit
5. Do you usually experience a feeling of incomplete bladder emptying?	◯ Yes<br>◯ No
If Yes, how much does it bother you?	◯ Not at all<br>◯ Somewhat<br>◯ Moderately<br>◯ Quite a bit
6. Do you ever have to push up on a bulge in the vaginal area with your fingers to start or complete urination?	◯ Yes<br>◯ No
If Yes, how much does it bother you?	◯ Not at all<br>◯ Somewhat<br>◯ Moderately<br>◯ Quite a bit
7. Do you feel you need to strain too hard to have a bowel movement?	◯ Yes<br>◯ No
If Yes, how much does it bother you?	◯ Not at all<br>◯ Somewhat<br>◯ Moderately<br>◯ Quite a bit
8. Do you feel you have not completely emptied your bowels at the end of a bowel movement?	◯ Yes<br>◯ No
If Yes, how much does it bother you?	◯ Not at all<br>◯ Somewhat<br>◯ Moderately<br>◯ Quite a bit
9. Do you usually lose stool beyond your control if your stool is well formed?	◯ Yes<br>◯ No
If Yes, how much does it bother you?	◯ Not at all<br>◯ Somewhat<br>◯ Moderately<br>◯ Quite a bit
10. Do you usually lose stool beyond your control if your stool is loose or liquid?	◯ Yes<br>◯ No
If Yes, how much does it bother you?	◯ Not at all<br>◯ Somewhat<br>◯ Moderately<br>◯ Quite a bit

11. Do you usually lose gas from the rectum beyond your control?	◯ Yes<br>◯ No
If Yes, how much does it bother you?	◯ Not at all<br>◯ Somewhat<br>◯ Moderately<br>◯ Quite a bit
12. Do you usually have pain when you pass your stool?	◯ Yes<br>◯ No
If Yes, how much does it bother you?	◯ Not at all<br>◯ Somewhat<br>◯ Moderately<br>◯ Quite a bit
13. Do you experience a strong sense of urgency and have to rush to the bathroom to have a bowel movement?	◯ Yes<br>◯ No
If Yes, how much does it bother you?	◯ Not at all<br>◯ Somewhat<br>◯ Moderately<br>◯ Quite a bit
14. Does part of your bowel ever pass through the rectum and bulge outside during or after a bowel movement?	◯ Yes<br>◯ No
If Yes, how much does it bother you?	◯ Not at all<br>◯ Somewhat<br>◯ Moderately<br>◯ Quite a bit
15. Do you usually experience frequent urination?	◯ Yes<br>◯ No
If Yes, how much does it bother you?	◯ Not at all<br>◯ Somewhat<br>◯ Moderately<br>◯ Quite a bit
16. Do you usually experience urine leakage associated with a feeling of urgency, that is, a strong sensation of needing to go to the bathroom?	◯ Yes<br>◯ No
If Yes, how much does it bother you?	◯ Not at all<br>◯ Somewhat<br>◯ Moderately<br>◯ Quite a bit
17. Do you usually experience urine leakage related to laughing, coughing, or sneezing?	◯ Yes<br>◯ No
If Yes, how much does it bother you?	◯ Not at all<br>◯ Somewhat<br>◯ Moderately<br>◯ Quite a bit

18. Do you usually experience small amounts of urine leakage (that is, drops)?	◯ Yes<br>◯ No
If Yes, how much does it bother you?	◯ Not at all<br>◯ Somewhat<br>◯ Moderately<br>◯ Quite a bit
19. Do you usually experience difficulty emptying your bladder?	◯ Yes<br>◯ No
If Yes, how much does it bother you?	◯ Not at all<br>◯ Somewhat<br>◯ Moderately<br>◯ Quite a bit
20. Do you usually experience pain or discomfort in the lower abdomen or genital region?	◯ Yes<br>◯ No
If Yes, how much does it bother you?	◯ Not at all<br>◯ Somewhat<br>◯ Moderately<br>◯ Quite a bit
Scale score POPDI	__________________________<br>((mean of answered Q1..Q6)x25)
Scale score CRADI-8	__________________________<br>((mean of answered Q7..Q14)x25)
Scale score UDI-6	__________________________<br>((mean of answered Q15..Q20)x25)
PFDI-20 Summary Score	__________________________<br>((sum of POPDI, CRADI-8, and UDI-6))

PISQ

Please complete the survey below.

Thank you!

Pelvic Organ Prolapse/Urinary Incontinence Sexual Function Questionnaire (PISQ)

Instructions: Following are a list of questions about you and your partner's sex life. All information is strictly confidential. Please check the box that best answers the question for you. While answering the questions, consider your sexuality over the past six months. Thank you for your help.

Which of the following best describes you:

- ◯ Not sexually active at all
- ◯ Sexually active with or without a partner

How frequently do you and your partner have sexual intercourse or activity?

- ◯ Every day
- ◯ 1 to 3 times a week
- ◯ 1 to 3 times a month
- ◯ Less than once a month
- ◯ Never

2. How frequently would you like to have sexual intercourse or activity?

- ◯ Every day
- ◯ 1 to 3 times a week
- ◯ 1 to 3 times a month
- ◯ Less than once a month
- ◯ Never

The following are a list of reasons why you might not be sexually active, for each one please indicate how strongly you agree or disagree with it as a reason that you are not sexually active.

	Strongly Agree	Somewhat Agree	Somewhat Disagree	Strongly Disagree
a) No partner	◯	◯	◯	◯
b) No Interest	◯	◯	◯	◯
c) Due to bladder or bowel problems (urinary or fecal incontinence) or due to prolapse (a feeling of or a bulge in the vaginal area)	◯	◯	◯	◯
d) Because of my other health problems	◯	◯	◯	◯
e) Pain	◯	◯	◯	◯

How much does the fear of leaking urine and/or stool and/or a bulging in the vagina (either the bladder, rectum or uterus falling out) cause you to avoid or restrict your sexual activity?

- ◯ Not at All
- ◯ A Little
- ◯ Some
- ◯ A Lot

Please select the number between 1 and 5 that best represents how you feel about your sex life.

Satisfied — Dissatisfied

(Place a mark on the scale above)

Please select the number between 1 and 5 that best represents how you feel about your sex life.

Adequate — Inadequate

(Place a mark on the scale above)

How strongly do you agree or disagree with each of the following statements:

	Strongly Agree	Somewhat Agree	Somewhat Disagree	Strongly Disagree
a) I feel frustrated by my sex life	◯	◯	◯	◯
b) I feel sexually inferior because of my incontinence and/or prolapse	◯	◯	◯	◯
c) I feel angry because of the impact that incontinence and/or prolapse has on my sex life	◯	◯	◯	◯

Overall, how bothersome is it to you that you are not sexually active?

◯ Not at all ◯ A Little
◯ Some ◯ A Lot

How often do you feel sexually aroused (physically excited or turned on) during sexual activity?

◯ Never
◯ Rarely
◯ Sometimes
◯ Usually
◯ Always

When you are involved in sexual activity, how often do you feel each of the following:

	Never	Rarely	Sometimes	Usually	Almost Always
Fulfilled	◯	◯	◯	◯	◯
Shame	◯	◯	◯	◯	◯
Fear	◯	◯	◯	◯	◯

How often do you leak urine and/or stool with any type of sexual activity?

◯ Never
◯ Rarely
◯ Sometimes
◯ Usually
◯ Always

Compared to orgasms you have had in the past, how intense are your orgasms now?

◯ Much less intense
◯ Less intense
◯ Same intensity
◯ More intense
◯ Much more intense

How often do you feel pain during sexual intercourse?

◯ Never
◯ Rarely
◯ Sometimes
◯ Usually
◯ Always

How often does your partner have a problem (lack of arousal, desire, erection, etc.) that limits your sexual activity?

◯ All of the time
◯ Most of the time
◯ Some of the time
◯ Hardly ever/rarely

In general, would you say that your partner has a positive or negative impact on each of the following:

	Very Positive	Somewhat Positive	Somewhat Negative	Very Negative
Your sexual desire	◯	◯	◯	◯
The frequency of your sexual desire	◯	◯	◯	◯

When you are involved in sexual activity, how often do you feel that you want more?

- ◯ Never
- ◯ Rarely
- ◯ Sometimes
- ◯ Usually
- ◯ Always

How frequently do you have sexual desire, this may include wanting to have sex, having sexual thoughts or fantasies, etc.?

- ◯ Daily
- ◯ Weekly
- ◯ Monthly
- ◯ Less often than once a Month
- ◯ Never

How would you rate your level (degree) of sexual desire or interest?

- ◯ Very high
- ◯ High
- ◯ Moderate
- ◯ Low
- ◯ Very low or none at all

How much does the fear of leaking urine, stool and/or a bulging in the vagina (prolapse) cause you to avoid sexual activity?

- ◯ Not at All
- ◯ A Little
- ◯ Some
- ◯ Lot

Please choose the number between 1 and 5 that best represents how you feel about your sex life.

Satisfied — Dissatisfied

(Place a mark on the scale above)

Please choose the number between 1 and 5 that best represents how you feel about your sex life.

Adequate — Inadequate

(Place a mark on the scale above)

Please choose the number between 1 and 5 that best represents how you feel about your sex life.

Confident — Not Confident

(Place a mark on the scale above)

How strongly do you agree or disagree with each of the following statements:

	Strongly Agree	Somewhat Agree	Somewhat Disagree	Strongly Disagree
I feel frustrated by my sex life	◯	◯	◯	◯
I feel sexually inferior because of my incontinence and/or prolapse	◯	◯	◯	◯

I feel embarrassed about my sex life	◯	◯	◯	◯
I feel angry because of the impact that incontinence and/or prolapse on my sex life	◯	◯	◯	◯

Patient Health Questionnaire Depression Scale (PHQ-9)

97) Have you ever been diagnosed with depression (including postpartum depression, Bipolar disorder or adjustment disorder)?
- ◯ No
- ◯ Yes
- ◯ I don't know

How often during the past 2 weeks were you bothered by....

	Notat all	Severaldays	More thanhalfthe days	Nearlyevery day
98) Little interest or pleasure in doing things	◯	◯	◯	◯
99) Feeling down, depressed, or hopeless	◯	◯	◯	◯
100) Trouble falling or staying asleep, or sleeping too much	◯	◯	◯	◯
101) Feeling tired or having little energy	◯	◯	◯	◯
102) Poor appetite or overeating	◯	◯	◯	◯
103) Feeling bad about yourself, or that you are a failure, or have let yourself or your family down	◯	◯	◯	◯
104) Trouble concentrating on things, such as reading the newspaper or watching television	◯	◯	◯	◯
105) Moving or speaking so slowly that other people could have noticed. Or the opposite--being so fidgety or restless that you have been moving around a lot more than usual	◯	◯	◯	◯
106) Thoughts that you would be better off dead or of hurting yourself in some way	◯	◯	◯	◯

107) If you checked off any problems, how difficult have these problems made it for you to do your work, take care of things at home, or get along with other people?
- ◯ Not difficult at all
- ◯ Somewhat difficult
- ◯ Very difficult
- ◯ Extremely difficult

108) PHQ9 Score ______________________

Generalized Anxiety Disorder 7 (GAD 7)

109) Have you ever been diagnosed with any form of anxiety?
○ No
○ Yes
○ I don't know

Over the last 2 weeks, how often have you been bothered by the following problems?

	Not at all	Several days	More than half the days	Nearly every day
110) Feeling nervous, anxious, or on edge	○	○	○	○
111) Not being able to stop or control worrying	○	○	○	○
112) Worrying too much about different things	○	○	○	○
113) Trouble relaxing	○	○	○	○
114) Being so restless that it's hard to sit still	○	○	○	○
115) Becoming easily annoyed or irritable	○	○	○	○
116) Feeling afraid as if something awful might happen	○	○	○	○

	Not difficult at all	Somewhat difficult	Very difficult	Extremely difficult
117) If you checked off any problems, how difficult have these made it for you to do your work, take care of things at home, or get along with other people?	○	○	○	○

118) GAD7 Score

____

NSESSS PTSD Short Scale

Please complete the survey below.

Thank you!

119) Have you ever been diagnosed with Post-traumatic stress disorder (PTSD)?

- ◯ No
- ◯ Yes
- ◯ I don't know

Instructions: People sometimes have problems after extremely stressful events or experiences. How much have you been bothered during the PAST SEVEN (7) DAYS by each of the following problems that occurred or became worse after an extremely stressful event/experience

**How much have you been bothered during the PAST SEVEN (7) DAYS by:**	Not at all	A little bit	Moderately	Quite a bit	Extremely
120) Having "flashbacks," that is, you suddenly acted or felt as if a stressful experience from the past was happening all over again (for example, you re-experienced parts of a stressful experience by seeing, hearing, smelling, or physically feeling parts of the experience)?	◯	◯	◯	◯	◯
121) Feeling very emotionally upset when something reminded you of a stressful experience?	◯	◯	◯	◯	◯
122) Trying to avoid thoughts, feelings, or physical sensations that reminded you of a stressful experience?	◯	◯	◯	◯	◯
123) Thinking that a stressful event happened because you or someone else (who didn't directly harm you) did something wrong or didn't do everything possible to prevent it, or because of something about you?	◯	◯	◯	◯	◯
124) Having a very negative emotional state (for example, you were experiencing lots of fear, anger, guilt, shame, or horror) after a stressful experience?	◯	◯	◯	◯	◯
125) Losing interest in activities you used to enjoy before having a stressful experience?	◯	◯	◯	◯	◯

126)

Being "super alert," on guard, or constantly on the lookout for danger?	◯	◯	◯	◯	◯
127) Feeling jumpy or easily startled when you hear an unexpected noise?	◯	◯	◯	◯	◯
128) Being extremely irritable or angry to the point where you yelled at other people, got into fights, or destroyed things?	◯	◯	◯	◯	◯

---

129) PTSD score

____
