# Supplementary material for: Pelvic floor and sexual health outcomes after cesarean hysterectomy for placenta accreta spectrum
Source: Pregnancy (Hoboken). 2025 Oct 18;1(6):e70134. doi: 10.1002/pmf2.70134 (PMC13344666; doi:10.1002/pmf2.70134)
Supplement: Supplementary file 2 [file PMF2-1-e70134-s002.docx]

|  | **Time from Surgery** | | |  |
| --- | --- | --- | --- | --- |
|  | **< 1 yr**  **n/N (%)** | **1 yr - 2 yrs**  **n (%)** | **>2 yrs**  **n(%)** | **P value^*^** |
| ≥1 symptom of pelvic organ prolapse | 5/15 (33.3) | 9/18 (50.0) | 6/31 (19.4) | 0.08 |
| Stress urinary Incontinence (SUI)^a^ | 3/14 (21.4) | 8/16 (50.0) | 13/31 (41.9) | 0.26 |
| Urge urinary incontinence (UUI)^a^ | 3/14 (21.4) | 4/16 (25.0) | 7/31 (22.6) | 1.00 |
| Incomplete emptying of the bladder | 2/15 (13.3) | 2/18 (11.1) | 4/31 (12.9) | 1.00 |
| Pelvic pain | 3/15 (20.0) | 7/18 (38.9) | 3/31 (9.7) | 0.05 |
| Avoid sex due to prolapse or incontinence^b^ | 1/10 (10.0) | 2/16 (12.5) | 1/26 (3.8) | 0.51 |
| Dyspareunia | 5/10 (50.0) | 6/16 (37.5) | 14/26 (53.9) | 0.58 |

Supplement 2.

*Chi-Square or Fisher’s Exact test where appropriate

^a^Among women with no history of incontinence (n=61)

^b^Among women reporting being sexually active (n=52)

^c^0=Never/Rarely, 1=Sometimes/Often/Always
